# Exploring the short‐term impact of swapping consumption from standard protein snacks to higher protein snacks on energy intake in social drinkers: Is protein worth a nudge?

**DOI:** 10.1002/fsn3.3902

**Published:** 2023-12-27

**Authors:** Alastair Kwok, Aimee L. Dordevic, Helen Truby

**Affiliations:** ^1^ Department of Nutrition, Dietetics and Food Monash University Notting Hill Victoria Australia; ^2^ School of Human Movement and Nutrition Sciences University of Queensland St Lucia Queensland Australia

**Keywords:** alcohol drinking, appetite, dietary proteins, eating, humans, snacks

## Abstract

Drinking alcoholic beverages stimulates food intake and contributes to the passive overconsumption of dietary energy. As protein is the most satiating of all the macronutrients, increased levels in snacks taken with alcohol have the potential to minimize excess energy consumption. We hypothesized that swapping consumption from retail‐available standard protein (SP) snacks to higher protein (HP) snack foods would increase satiety and reduce acute food energy intake in social drinkers. A randomized single‐blind crossover trial with 19 healthy participants aged 19–31 years was conducted. Participants attended two separate testing sessions, where they ingested white wine (30 g alcohol) and were offered ad libitum access to either HP snacks with a protein‐fortified dip or SP snacks with a dip. There were no significant differences in mean food mass, food energy intake, or subjective appetite ratings between the high and SP snacks (all *p* > .05). Mean protein intake was significantly increased with HP snacks compared with standard snacks (*p* < .001). Plasma glucose median incremental area under the curve and mean peak were significantly higher with the SP snacks (all *p* < .05) but remained within the reference range. This study demonstrated that consumption of a higher amount of protein after a moderate alcohol dose does not result in a change in food mass and energy intake or promote satiety in healthy young adults. The potential for a simple swap to different snack types is unlikely to bring substantial benefits to social drinkers and reduce passive energy consumption.

## Graphical abstract

In this research, the short‐term impact of a swap in consumption from standard protein (SP) snacks to higher protein (HP) snacks following alcohol consumption was explored in 19 healthy young adult social drinkers. Acute food energy intake and satiety were similar between the snacks, although plasma glucose median incremental area under the curve and mean peak were higher in the SP snacks compared with the HP snacks. A simple swap in snack consumption following alcohol consumption had minimal benefit in healthy young adult social drinkers; its acute impact is worthy of further research in those with impaired glucose control.

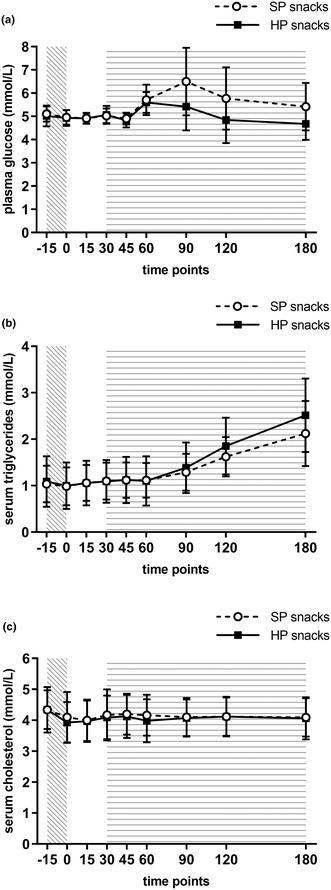

## 1 INTRODUCTION

Although alcoholic beverages are commonly consumed worldwide, consumption levels changed with the onset of the COVID‐19 pandemic, modulated by region, government restrictions, and time (Acuff et al., 2022; Roberts et al., 2021). In Australia, the use of alcoholic beverages is widespread and continues to play a substantial part in the culture. In 2020–2021, 66.4% of Australian adults reported their alcohol consumption did not change, whereas 23.9% decreased consumption and 9.8% increased consumption when compared with 12 months earlier (Australian Bureau of Statistics, 2022).

The energy density of alcohol (29 kJ/g) is second only to fat (37 kJ/g) and may contribute to positive energy balance, leading to weight gain. The majority of Australian adults (67.0%) are now above a healthy weight (Australian Bureau of Statistics, 2018), a key risk factor for developing chronic diseases such as cardiovascular disease and diabetes (World Health Organization, 2014). The relationship between alcohol consumption and body weight remains elusive but is potentially mediated by biological sex and alcohol type (Bendsen et al., 2013; Sayon‐Orea et al., 2011; Traversy & Chaput, 2015).

One impact of alcohol consumption is stimulation of appetite, which leads to an increased urge to snack (Gough et al., 2021; Rose et al., 2015; Traversy & Chaput, 2015; Yeomans, 2010). Reported mechanisms include evidence that alcohol disrupts appetite signals, induces the release of endogenous opioids that increase the orosensory reward or palatability of foods (Widdowson & Holman, 1992; Yeomans, 2010; Yeomans et al., 2003; Yeomans & Gray, 2002), and binds to type‐A gamma‐aminobutyric acid (GABAa) receptors, stimulating GABAa neurotransmission, which enhances food intake in animals (Lobo & Harris, 2008; Yeomans et al., 2003). Individuals who have disinhibited eating behaviors may be more responsive to snacking after a small dose of alcohol in a social setting (Rose et al., 2015).

Our recent systematic review demonstrated that energy intake from alcohol was not compensated for in the short term by consuming less energy from other dietary sources (Kwok et al., 2019). Therefore, energy intake from alcohol is likely to be additive to other dietary source, including snacks, particularly with regular alcohol consumption, and lead to passive overconsumption of dietary energy.

Protein is generally accepted as the most satiating macronutrient (Halton & Hu, 2004; Westerterp‐Plantenga et al., 2009). In countries such as Great Britain and Australia, alcohol is commonly consumed with snack foods, which are typically low in protein (National Health and Medical Research Council, 2013; Warde et al., 2023). In an Australian study, chips, crisps, and extruded snacks were shown to contain the highest median energy, fat, and saturated fat content of all the available snack foods reviewed and had a low median protein content of 4.4 g (interquartile range 0.9 g) per median serving of 50 g (interquartile range 5 g) (Walker et al., 2008). Substitution of carbohydrates or fats with protein is reported to promote satiety (Ortinau et al., 2014; Tannous dit El Khoury et al., 2006). As such, increasing the protein content of snack foods consumed alongside alcoholic beverages may promote satiety, and lead to reduced total food energy consumption when compared with standard snacks. Therefore, macronutrient distribution may inform the selection of snacks for consumption, where snacks with a different macronutrient distribution may be a strategy to influence appetite and satiety in social drinkers. There is considerable interest in “nudging strategies” that subtly influence consumers toward healthier food behaviors, especially in social settings (Bucher et al., 2016; Hollands et al., 2019; Wilson et al., 2016).

This study explored whether simply swapping consumption from retail‐available standard protein (SP) snacks to higher protein (HP) snacks had the potential to alter short‐term food energy intake and satiety after a standardized moderate dose of alcohol (white wine). Secondary outcomes included assessing the acceptability of the HP snacks and the short‐term impact of consuming the different snacks and their macro‐nutrient composition on postprandial biochemical responses. It was hypothesized that consumption of HP snacks could elicit a greater satiety response and decrease food energy intake compared with SP snacks after a moderate dose of alcohol.

## 2 MATERIALS AND METHODS

### 2.1 Study design

A single‐blind, randomized crossover design was used. Eligible participants were randomly allocated into two groups to consume first either the SP snacks or HP snacks, with a 1:1 allocation ratio. All participants attended on two separate days, separated by an interval of at least 1 week.

### 2.2 Participant recruitment, selection, and randomization

Participants were healthy adults with a body mass index between 18.5 and 30 kg/m^2^ with non‐restrained eating behavior and not dieting to lose weight. The inclusion criteria were aged 18–65 years; usual consumers of alcoholic beverages with an average between 10 and 210 g of ethanol per week.

Exclusion criteria for the study were: Dutch Eating Behavior Questionnaire restrained subscale score >2.5 (van Strien et al., 1986); Alcohol Use Disorder Identification Test overall score ≥16 and/or a dependence score ≥4 (Babor et al., 2001); type 1 diabetes or type 2 diabetes; pregnant or breastfeeding; food allergies or gluten intolerance; severe dietary restrictions such as veganism; smoker; abstain or consume excessive levels of alcohol (consume >60 g of ethanol on seven or more occasions in the preceding month).

Participants were recruited through social media advertisements and advertisement flyers displayed around the university. Eligible participants were randomized into one of the two study arms using a computerized random number generator by a researcher external to the study. The allocation sequence was concealed in opaque envelopes from the investigator (AK). Participant enrolment, data collection, and statistical analysis of results were conducted by the investigator (AK). Participants were informed that the purpose of the study was to investigate the effects of drinking a moderate amount of alcohol on the taste and acceptability of two different sets of food snacks. Consistent with the bogus taste test measure, the true purpose of the study was not initially revealed, as this could have influenced the participants' eating behavior (Robinson et al., 2017).

### 2.3 Ethics

The study was conducted according to the guidelines established in the Declaration of Helsinki. All procedures involving human participants were approved by the Monash University Human Research Ethics Committee (2017‐0383‐12265). Written informed consent was obtained from all participants. The trial was registered with the Australian New Zealand Clinical Trials Registry (ACTRN12617000136303).

### 2.4 Intervention

A brief review of the savory snacks readily available from Australian retailers was conducted to inform the selection of the two HP snacks and two SP snacks. The HP snacks were bean and brown rice crisps with a protein content of 17.8 g protein/100 g plus black sesame brown rice crackers with a protein content of 14 g protein/100 g. A flavored dairy dip fortified with whey protein isolate was provided with the HP snacks (15 g protein/100 g). The SP snack comparators were potato crisps with a protein content of 7.6 g protein/100 g plus cassava crisps with a protein content of 1.2 g protein/100 g. A flavored dairy dip with the same flavor as the dip that accompanied the HP snacks was provided. Nutrition information for the selected HP snacks and SP snacks and flavored dips is presented in Table 1.

**TABLE 1:** Nutritional information of standard protein (SP) snacks, higher protein (HP) snacks, and flavored dairy dips (per 100 g).

	SP snacks		HP snacks		Flavored dairy dips						
	Cassava crisps – Original flavor	Potato crisps – Sea salt flavor	Black sesame rice crackers	Bean and rice crisps – Sea salt flavor	Avocado	Baby spinach and feta	French onion	Garlic hummus	Guacamole	Hummus	Spicy capsicum
Energy (kJ)	1950	2090	1965	2100	1230	1540	1160	1310	769	970	841
Energy density (kJ/g)	19.5	20.9	19.7	21	12.3	15.4	11.6	13.1	7.69	9.7	8.41
Protein (g)	1.2	7.6	14	17.8	6	8	5.6	6.7	2.5	6.6	4.8
Fat (g)	18.8	27	23.4	15.9	28.9	36.1	26.7	25.2	15	16.2	15.6
Saturated fat (g)	1.6	2.6	3.5	1.2	16.6	13.2	14.7	2.4	5.7	1.4	8.8
Total Carbohydrates (g)	67.3	56.6	46.5	64	3.6	3.7	4.3	13.4	9.9	15.2	10.7
Available Carbohydrates (g)	63.1	54.5	37.5	42.6	N/A	N/A	N/A	N/A	N/A	N/A	N/A
Sugar (g)	7.3	0	1.2	1.2	2	2.7	2.8	3.8	3.7	0.4	1.9
Dietary fiber (g)	4.2	2.1	9	21.4	N/A	N/A	N/A	N/A	N/A	N/A	N/A
Sodium (mg)	560	488	427	339	379	443	725	269	609	370	610

*Note*: 200 g of the flavored dairy dip is served alongside the bowl of SP snacks. 30 g of whey protein isolate is added to the 200 g of the flavored dairy dip and then served alongside the bowl of HP snacks. The dietary fiber content of the flavored dairy dips was not available, despite requesting this information from the dip manufacturers.

Abbreviation: N/A, not applicable.

All snacks were presented in bowls in 150 g amounts, and dips were presented in bowls in 200 and 230 g (when fortified with whey protein isolate) amounts. They were presented without any labeling as participants were blinded to the snack allocation. The bean and rice crisps were matched with the cassava crisps within 8% for percentage energy from total carbohydrates and for percentage energy from fat. There was a 21% difference in the percentage energy from available carbohydrates between the snacks. The black sesame rice crackers were matched with the potato crisps within 6% for percentage energy from total carbohydrates and percentage energy from fat. There was a 12% difference in percentage energy from available carbohydrates between the snacks.

To avoid over‐ or under‐consumption due to the presence of highly desirable foods or unpalatable foods, respectively, participants’ liking for snack flavors and dip flavors was recorded. Prior to the first testing session, participants were asked to rank their five most favorite and five least favorite crisp snacks and their flavor profiles, and their five most favorite and five least favorite dip flavors. Based on the participants' responses to the questionnaire, the snack buffets consisted of either the fourth flavor preference or the fifth flavor preference that was available for the two selected SP snacks and two HP snacks, as rated by each participant. If the SP snacks and HP snacks were not available in those flavors, the plain flavor of the snack was provided instead. Provision of moderately liked flavor profiles was used to encourage participants to consume both snacks provided for each condition rather than consume a preferred flavored snack in a greater quantity than the other (Blundell et al., 2010).

### 2.5 Experimental protocol and measurements

**FIGURE 1:**
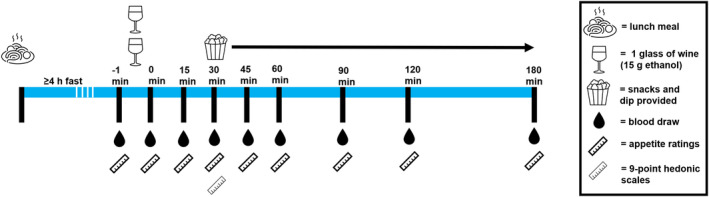
Testing session procedure.

Participants were instructed to refrain from participating in strenuous exercise and from consuming alcohol for 24 h prior to each testing day. On the morning of each testing session day, participants were instructed to eat their usual breakfast. They then consumed a standardized commercially prepared lunch meal, either 400 g beef lasagna (1630 kJ, 22.8 g protein, 9.2 g total fat, 49.2 g carbohydrates, 7.6 g dietary fiber) or 400 g vegetarian lasagna (1550 kJ, 20 g protein, 4.8 g total fat, 57.2 g carbohydrates, 7.6 g dietary fiber), between 1100 and 1200 h and then fasted for 4 h and attended the laboratory (Figure 1). For the preparations leading up to each of the two testing session days, the instructions and the standardized lunch meal provided were identical for each participant.

Testing commenced at the Be Active Sleep Eat Facility at Monash University, Australia, a purposefully designed participant‐friendly space, from 1600 h and lasted for 240 min. Participants were tested together and were seated at their own tables in the main laboratory area. They were instructed to bring a book, laptop, or phone to engage them during the testing session, and they could also socialize with other participants if they wished. An intravenous cannula was placed in the arm by a trained phlebotomist, a baseline blood sample was collected, and appetite ratings were recorded using visual analogue scales (VAS). Participants then consumed two glasses (292 mL) of white wine, which contained 30 g of ethanol, within 15 min. Immediately following (t0), blood sampling and VAS were collected again at 15, 30, 45, 60, 90, 120 and 180 min.

At 30 min after alcohol consumption, participants were provided with either SP or HP snacks to consume ad libitum for the remainder of the testing session (i.e., between the 30 and 180 min period). Snacks were automatically refilled when the bowls were near empty or empty. In order to distract participants from the main purpose of the study, they completed a 9‐point hedonic scale for flavor, texture, mouthfeel, appearance and overall liking for the snacks and dip (Peryam & Pilgrim, 1957). A rating of “like slightly” or more was considered to have liked the specific attribute for the snack and dip, whereas a rating of “dislike slightly” or less was considered to have disliked the specific attribute for the snack and dip. Participants were permitted to leave when their breath alcohol concentration was measured with a breathalyzer (AlcoLimit Breathalyzers, Manly Vale, Australia) to be 0.02% or below. This session was repeated with the alternate snacks at least 1 week later.

Participants were provided with a written debrief by email after the data analysis had been completed. A written debrief was provided to participants, with the opportunity to contact the authors with any queries. There was no record of participants questioning the cover story, either during the testing sessions or after the written debrief was circulated.

### 2.6 Measurements

All snacks and dips were weighed using a digital food scale before being provided to the participant and re‐weighed when they were refilled or at the end of each testing session. The snacks and dips were discreetly weighed in a separate area from where the participants were seated during the testing session. Food mass intake was then calculated by subtracting the post‐testing weights from the pre‐testing session weights, including any refills.

Appetite VAS consisted of hunger, fullness, and desire to eat ratings. The VAS were conducted on a Qualtrics survey (Qualtrics, Provo, Utah, USA) with a computerized line between 0 and 100. The questions were delivered to participants on an iPad Air (Apple, Cupertino, California, USA).

At each time point, a blood sample of 7.5 mL was collected from participants and stored in a 4 mL ethylenediaminetetraacetic acid (EDTA) tube and a 3.5 mL serum separator tube. The plasma samples from the EDTA tubes were centrifuged at 590×*g* at 4°C for 12 min in the Eppendorf Centrifuge 5702R (Eppendorf, Hamburg, Germany). Serum samples from the serum separator tubes were allowed to clot for 30 min and then centrifuged at 1700×*g* at 22°C for 10 min. Samples were stored at −80°C until analysis. Plasma glucose (Ref 981779), plasma ethyl alcohol concentration (Ref 10016397), serum total cholesterol (Ref 981813) and serum triglycerides (Ref 981786) were measured as per the manufacturer's instructions using the Indiko Clinical and Specialty Chemistry analyzer (Thermo Fisher Scientific, Waltham, Massachusetts, USA). All samples were analyzed in duplicate; any sample with a coefficient of variation difference of more than 5% was re‐run. The intra‐assay coefficient of variation for glucose, total cholesterol, triglycerides, and ethyl alcohol concentration was <2%.

### 2.7 Data and statistical analysis

Twenty subjects per group were deemed sufficient to detect differences in food energy intake between the groups based on previous studies (Caton et al., 2005, 2007; Hetherington et al., 2001).

Data are presented as the mean (standard deviation [SD]) unless otherwise specified. Food weights were converted to food energy intake based on the manufacturers’ nutrition information panel for the snacks and dip. The total area under the curve (tAUC) for hunger, fullness, desire to eat ratings, and plasma alcohol concentration were calculated using the trapezoidal rule. The incremental area under the curve (iAUC) for plasma glucose, serum total cholesterol, and serum triglycerides was calculated using the trapezoidal rule. Statistical testing for between‐group comparisons involved detecting evidence of carryover effects and differences between treatment effects (Wellek & Blettner, 2012). Testing to confirm the negligible carryover effect was conducted on the within‐subject sums of the results from the two testing periods with either an independent samples *t*‐test or a Mann–Whitney rank sum test, depending on the data distribution. The testing for the differences on all outcome measures was conducted between the two groups, which were the group that received one treatment first and the group that received the other treatment first. Independent samples *t*‐test or a Mann–Whitney rank sum test were used on the within‐subject differences of the results from period 1 and period 2, as applicable based on data distribution (Wellek & Blettner, 2012). Cohen's *d* (*d*) effect sizes were calculated using Psychometrica (Lenhard & Lenhard, 2022). They can be cautiously interpreted as follows: small, 0.2 to 0.49; medium, 0.50 to 0.79; large, 0.80 to 1.29; and very large, 1.30 or more (Ellis, 2010).

All statistical analyses were performed using the Statistical Package for Social Sciences software (IBM SPSS version 25, IBM Corp., Armonk, NY, USA). A value of *p* < .05 was considered statistically significant. No correction for multiple testing was used, as multiple usages of the *t*‐test were used where the results of the individual test were deemed important (Armstrong, 2014).

## 3 RESULTS

### 3.1 Participants

Of the 246 people who expressed their interest in participating, 175 did not meet the eligibility criteria, whereas 61 chose not to proceed to a screening session. A total of 20 healthy adults were recruited, and the study was completed prior to the onset of the COVID‐19 pandemic in early 2020. Nineteen participants completed the study; one participant dropped out after randomization and no data were collected. One participant only had blood collected from one testing session due to difficulty with cannulation, so markers were excluded from analysis. Baseline characteristics of the 19 participants are presented in Table 2. The age range of the 19 participants was 19–31 years and the BMI range was 19.0–26.8 kg/m^2^. The weight range was 46.3–85 kg and the usual alcohol consumption range was 1–10 standard drinks per week.

**TABLE 2:** Participant baseline characteristics.

	Value
Sex, *n* (%)	
Male	8 (42)
Female	11 (58)
Ethnicity, *n* (%)	
Caucasian	8 (42)
Asian	11 (58)
Age, years	22.4 (2.9)^a^
Weight, kg	63.5 (9.2)
Height, m	1.7 (0.1)
Body mass index, kg/m^2^	22.5 (2.4)
Usual alcohol consumption, standard drinks per week	2.2 (4.0)^a^

*Note*: Mean (SD) values or n (percentage) are shown.

^a^ Median (interquartile range) reported.

### 3.2 Food energy and macronutrient intake

There were no differences in the consumption of mean food mass (*p* = .88) or mean food energy intake (*p* = .28) between HP and SP snacks (Table 3). The mean protein intake was higher with HP snacks (mean difference: 33.5 g protein, 559 kJ) than with SP snacks (*p* < .001). No differences were observed between the snacks for mean fat intake (HP: 55.6 g ± SD 18.5 g and SP: 59.3 g ± 20 g, *p* = .25) and mean total carbohydrate intake (HP: 105 g ± 33.9 g and SP: 111 g ± 33.8 g, *p* = .38). However, the mean available carbohydrate intake was significantly higher with the SP snacks (mean difference: 28.2 g of available carbohydrates, 479 kJ, *p* < .001). Sub‐analyses for males and females were consistent with total group results except for mean fat intake in males, which was higher with SP snacks than with HP snacks (*p* = .03).

**TABLE 3:** Food energy and macronutrient intake for standard protein (SP) and higher protein (HP) snacks.

	SP snacks and dip			HP snacks and protein‐fortified dips			*p*‐Value for differences between snacks	*d*
	Mean	SD	Range	Mean	SD	Range		
Food mass intake (g)—all	285.9	85.4	112–408	289.5	87.7	114–432	.88	0.07
Food mass intake (g)—males	322.5	62.4	234–392	297.1	70.5	193–418	.15	1.16
Food mass intake (g)—females	259.3	92.6	112–408	284	101.4	114–432	.31	0.65
Food energy intake (kJ)—all	4343	1297	1512–6738	4664	1402	1772–7192	.28	0.05
Food energy intake (kJ)—males	4810	846	3196–5873	4772	1109	3044–6904	.91	0.08
Food energy intake (kJ)—females	4004	1492	1512–6738	4585	1632	1772–7192	.16	0.92
Protein intake (g)—all	12.9	4.8	5.8–22.6	46.4	13.8	19.4–72.8	<.001	12.09
Protein intake (g)—males	13.5	3.1	8.8–16.7	46.1	10.2	29.8–64.4	<.001	8.25
Protein intake (g)—females	12.5	5.9	5.8–22.6	46.6	16.4	19.4–72.8	<.001	5.27
Fat intake (g)—all	59.3	20	19.9–105.3	55.6	18.5	26–102.6	.25	0.54
Fat intake (g)—males	63.6	11.8	44.1–76.4	54.3	11.9	35.8–72.5	.03	2.05
Fat intake (g)—females	56.1	24.4	19.9–105.3	56.5	22.8	26–102.6	.92	0.07
Total carbohydrate intake (g)—all	111.7	33.8	39.0–162.8	105.7	33.9	30.4–168.9	.38	0.42
Total carbohydrate intake (g)—males	129.3	25.7	81.8–162.8	114.9	28.7	73.1–168.5	.21	1.04
Total carbohydrate intake (g)—females	98.9	34.1	39–140.8	98.9	37	30.4–168.9	.83	0.14
Available carbohydrate intake (g)—all	106.3	32.2	37.1–154.7	78.1	78.1	21.4–124.6	<.001	2.11
Available carbohydrate intake (g)—males	123.3	24.0	77.9–154.7	84.9	20.9	53.8–124.6	.005	3.11
Available carbohydrate intake (g)—females	94.0	32.5	37.0–134.6	73.2	26.6	21.4–119.1	.01	1.84

Abbreviation: *d*, Cohen's *d*.

### 3.3 Appetite ratings and biochemical measures

**FIGURE 2:**
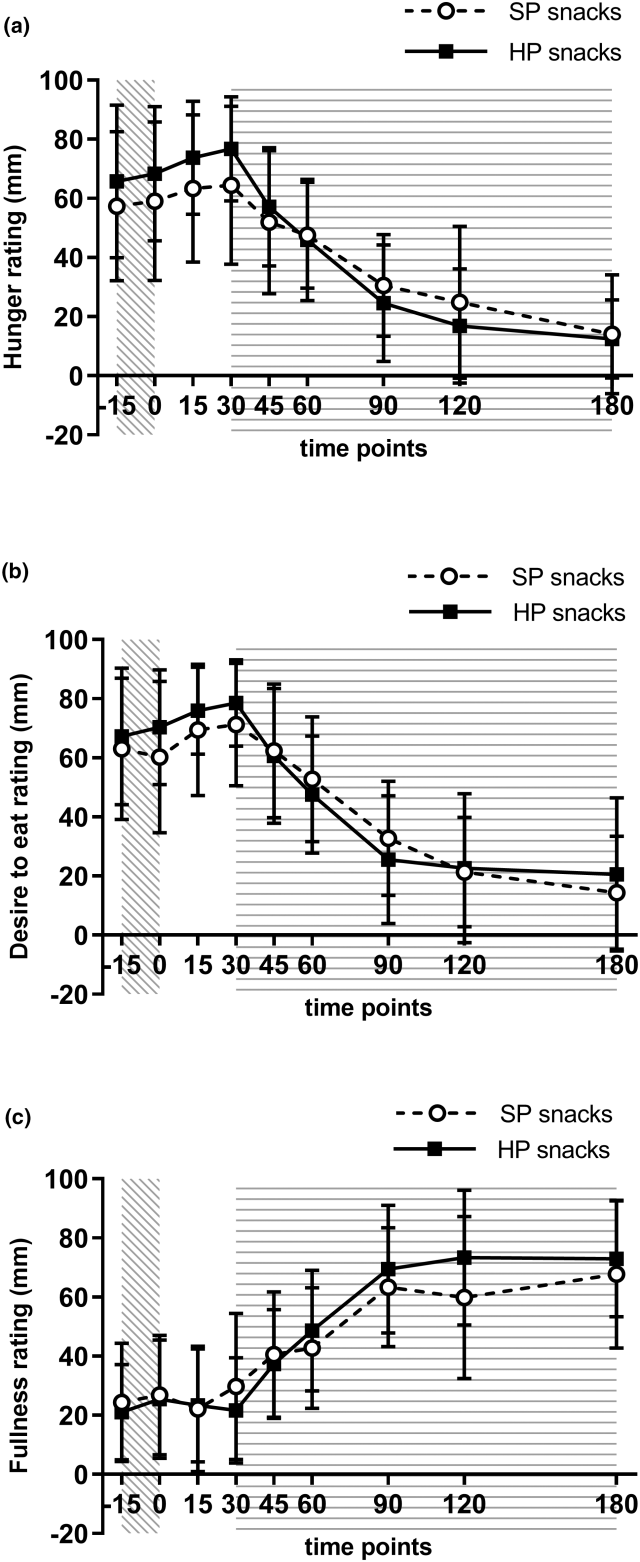
Trajectory of hunger ratings during testing session (a), trajectory of desire to eat rating during testing session (b), and trajectory of fullness ratings during testing session (c). Values are means with a standard deviation. 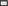: alcohol consumption period; 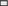: snack provision period.

**FIGURE 3:**
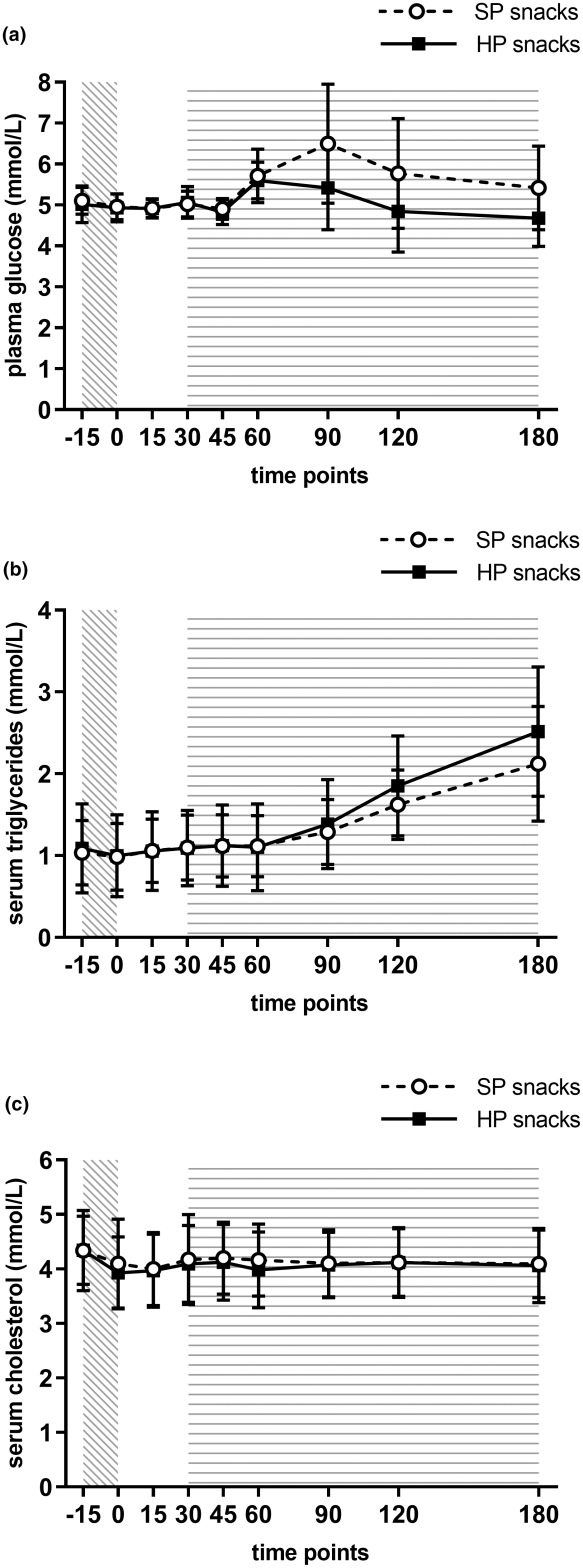
Trajectory of plasma glucose response (a), trajectory of serum triglyceride response (b), and trajectory of serum cholesterol response (c). Values are means with a standard deviation.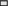: alcohol consumption period;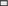: snack provision period.

**FIGURE 4:**
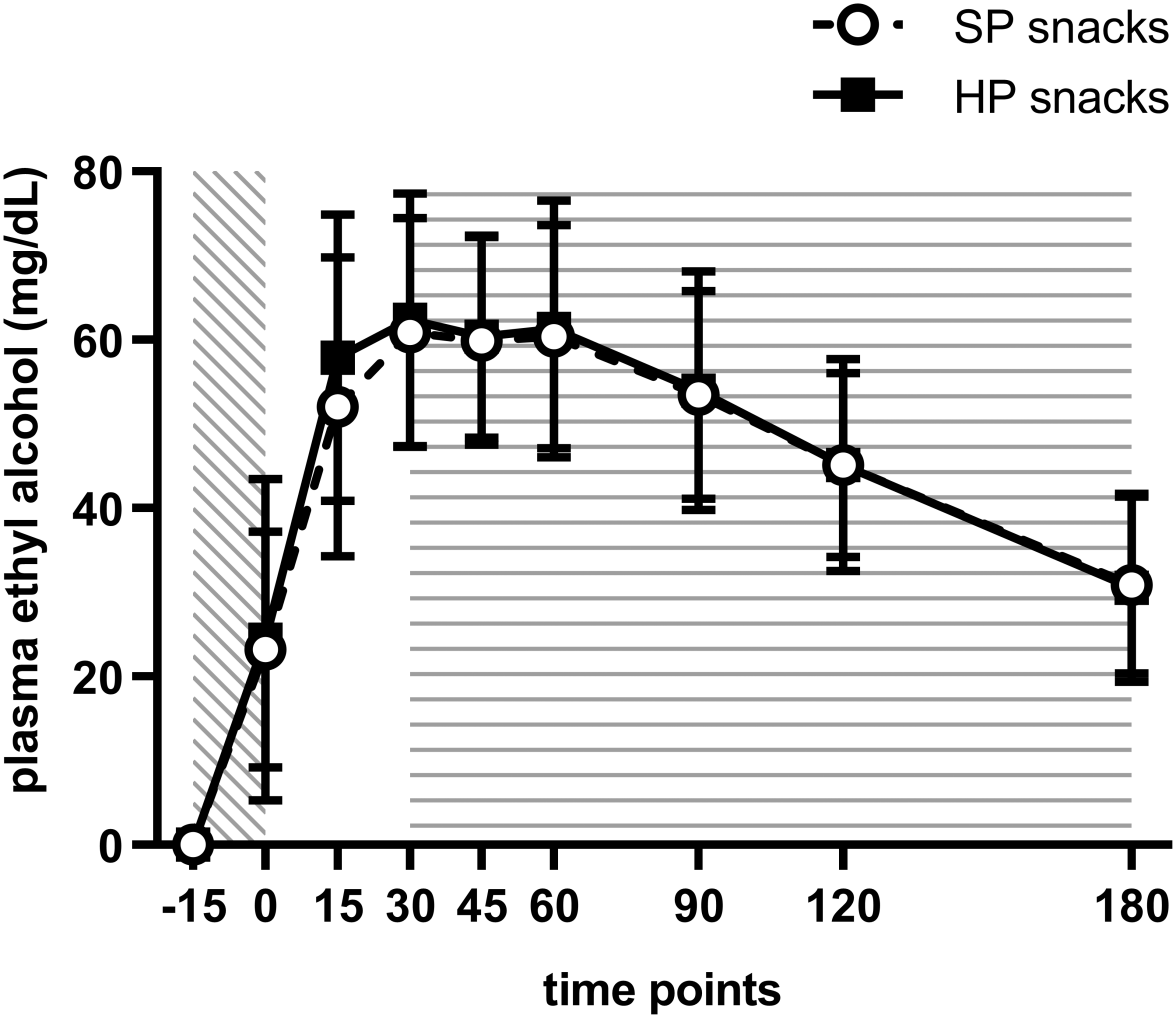
Trajectory of plasma alcohol response. Values are means with a standard deviation.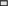: alcohol consumption period;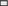:snack provision period.

Appetite ratings and biochemical measures are presented in Table 4. At baseline, no differences were observed between the HP and SP snacks for mean hunger (HP: 65.7 ± SD 25.8 and SP: 57.3 ± SD 26.0, *p* = .19, *d* = 0.65), mean desire to eat (HP: 65.6 ± SD 22.7 and SP: 63.1 ± SD 24.7, *p* = .50, *d* = 0.70), and mean fullness ratings (HP: 22.2 ± SD 15.7 and SP: 24.3 ± SD 20.6, *p* = .17, *d* = 0.32). No differences were observed between the HP and SP snacks for median hunger tAUC (*p* = .97, Figure 2a), mean desire to eat tAUC (*p* = .67, Figure 2b), and mean fullness tAUC (*p* = .17, Figure 2c). The mean peak plasma glucose concentration (*p* = .004, Figure 3a) and median plasma glucose iAUC (*p* = .006) were significantly higher with SP snacks compared with HP snacks. The mean serum triglycerides peak concentration was significantly higher with the HP snacks than the SP snacks (*p* = .02, Figure 3b). There were no differences between the snacks for serum cholesterol measures (Figure 3c), fasting plasma glucose, serum triglycerides (fasting and iAUC), and plasma alcohol concentration measures (Figure 4).

**TABLE 4:** Subjective appetite ratings and biochemical measures for standard protein (SP) and higher protein (HP) snacks.

	SP snacks and dips		HP snacks and protein‐fortified dips		*p*‐Value for differences	*d*
	Mean	SD	Mean	SD		
Hunger tAUC (mm × 195 min)^a^	7298	4695	7290	2993	.97	0.04
Desire to eat tAUC (mm × 195 min)	8453	3013	8764	2109	.67	0.20
Fullness tAUC (mm × 195 min)	9742	2749	10,600	2417	.17	0.66
Fasting glucose concentration (mmol/L)	5.08	0.45	4.92	0.46	.29	0.45
Peak glucose concentration (mmol/L)	6.86	1.15	5.98	0.58	.004	1.60
Glucose iAUC (mmol/L × 195 min)^a^	120.85	169.14	35.74	60.57	.006	1.58
Fasting triglycerides concentration (mmol/L)	1.04	0.4	1.09	0.55	.61	0.25
Peak triglycerides concentration (mmol/L)	2.14	0.68	2.52	0.79	.02	1.20
Triglycerides iAUC (mmol/L × 195 min)	76.09	40.72	90.34	29.42	.21	0.62
Fasting cholesterol concentration (mmol/L)	4.34	0.74	4.34	0.63	.99	0.006
Peak cholesterol concentration (mmol/L)	4.46	0.75	4.4	0.65	.62	0.22
Cholesterol iAUC (mmol/L × 195 min)^a^	0.02	2.2	0	1.58	.93	0.04
Peak ethyl alcohol concentration (mmol/L)	66.28	13.03	68.26	14.79	.5	0.31
Ethyl alcohol tAUC (mg/dL × 195 min)	8860	1977	9021	2208	.58	0.27

Abbreviations: *d*, Cohen's *d*; iAUC, incremental area under the curve; tAUC, total area under the curve.

^a^ Median and interquartile range reported.

### 3.4 9‐point hedonic scales

The 9‐point hedonic scale ratings for flavor, texture, mouthfeel, appearance, and overall liking of the SP and HP snacks are presented in Table 5. The median scores for the SP snacks ranged between 7 and 8 for flavor, texture, mouthfeel, appearance, and overall liking. Of all participants, the overall liking of the SP potato crisps and the SP cassava crisps was 84% and 89%, respectively. The median 9‐point hedonic scale scores for the HP snacks were lower than the SP snacks. They ranged between 4 and 7 for the black sesame rice crackers, whereas they ranged between 6 and 7 for the bean and rice crisps. For the overall liking of the HP snacks, there was a preference for the bean and rice crisps (84%), compared with the black sesame rice crackers (42%). This was reflected in the median scores for flavor, texture, mouthfeel, appearance, and overall liking, which ranged from 6 to 7 for the HP bean and rice crisps, whereas they ranged from 4 to 7 for the HP black sesame rice crackers. For the overall liking of the dips, all participants liked the standard dip, whereas 95% of participants liked the fortified protein dip. The median scores for the 9‐point hedonic scales were similar between the standard dip and the fortified protein dip.

**TABLE 5:** 9‐point hedonic scale ratings for the standard protein (SP) and higher protein (HP) snacks.

	SP snacks and dip						HP snacks and dip					
	Cassava crisps		Potato crisps		Dip		Black sesame rice crackers		Bean and rice crisps		Protein‐fortified dip	
	Median	IQR	Median	IQR	Median	IQR	Median	IQR	Median	IQR	Median	IQR
Flavor	8	2	8	1	8	1	5	2	7	2	8	1
Texture	8	2	8	1	8	1	7	2	7	1	8	1
Mouthfeel	8	2	7	1	8	0	5	2	7	2	8	2
Appearance	7	2	7	2	8	2	4	2	6	3	7	3
Overall liking	8	1	8	1	8	0	5	2	7	2	8	1

## 4 DISCUSSION

This study explored the impact of a simple swap from retail‐available SP snacks to HP snacks on short‐term food energy intake and subjective appetite ratings after the consumption of two glasses of wine in healthy young adults. The acceptability of consuming HP snacks and the short‐term impacts of snack consumption on biochemical measures were also explored. These findings suggest that swapping to HP snacks may not have a substantial impact on reducing food energy intake or increasing satiety responses in young adults. Participants preferred the SP snacks compared with the HP snacks, although one HP snack was more acceptable than the other HP snack. The finding that HP snacks elicited a lower glucose response in this group of healthy adults would suggest that a “snack swap” strategy would be worthy of further evaluation in people with impaired glucose metabolism.

HP snacks did not result in reduced food energy intake in the short term or increased feelings of fullness. It was expected that HP snacks would elicit a greater satiety response, as the mean protein intake was 33.5 g higher for HP snacks compared with SP snacks. This mean difference is slightly more than 50% of the Australian recommendations for daily protein for a 75‐kg adult male (National Health and Medical Research Council, 2006). There are inconsistencies reported for the ideal level of protein intake required to promote satiety. Healthy women consuming an afternoon snack of yoghurt with 24 g of protein demonstrated a reduction in hunger, increased fullness, and delayed subsequent eating compared with isoenergetic yoghurt with either 5 g or 14 g of protein content (Douglas et al., 2013). However, three isoenergetic savory snacks in the afternoon (45.1 g, 7.1 g and 5.8 g of protein) did not delay the request for dinner and differ in ad libitum energy intake at dinner in males (Marmonier et al., 2000). Despite the present study demonstrating a higher mean protein intake difference between the snacks, no differences in food intake were detected as a total group or when analyzed by sex. This strongly suggests that a simple “nudge” to change social drinkers to snack on a HP snack item would be unlikely to result in a substantial reduction in food energy intake. As this is the first study to our knowledge to compare the effects of swapping consumption from commercial SP snacks to HP snacks on satiety after alcohol consumption, the effects of consuming an alcoholic beverage may override the expected satiating properties of the HP snacks.

Alcohol consumption may influence the amount of snacks consumed, regardless of their macronutrient composition. A learned response may exist between alcohol and high‐fat and salty savory foods, which are commonly consumed together in Western countries (Caton et al., 2004, 2005; Schrieks et al., 2015). Although Caton et al. (2004) demonstrated increased intake of crisps after consuming 32 g compared with 8 g alcohol (from beer), there were no differences in intake of crisps between 8 g alcohol and a non‐alcoholic beer. In a study conducted on heavy social drinkers, Adams and Wijk (2020) found that the amount of salty crisps consumed over a 15‐min period was similar following consumption of either vodka or a non‐alcoholic beverage. In contrast, Schrieks et al. (2015) reported that vodka increased the intake and liking of high‐fat savory foods compared with a non‐alcoholic beverage. In studies where alcohol consumption was observed to increase energy intake, this was often driven by the intake of fat and protein (Cummings et al., 2019). As the fat content of the HP and SP snacks was similar in the present study, alcohol consumption may have increased the intake and liking of these snacks to a similar extent. The participants' preference and higher reported 9‐point hedonic scale ratings for the SP snacks did not deter them from consumption of the HP snacks. This effect may have been partly driven by the proposed mechanisms of alcohol on regulation of food intake. However, it is possible that participant activities during testing sessions, such as socialization and the use of electronic devices, may have influenced snack choice and intake (Cruwys et al., 2015; Tabares‐Tabares et al., 2022).

The increased plasma glucose peak and iAUC observed with SP snacks may be attributed to both protein and actions on decreasing postprandial glycemia and the higher available carbohydrate content in the snacks (Akhavan et al., 2014; Kashima et al., 2016). Previous research is conflicted on the effects of alcohol on postprandial blood glucose in healthy individuals, which is likely due to differences in study design, type of alcoholic beverage, and meal used (Hatonen et al., 2012; Middlemiss et al., 2007). However, the addition of protein to carbohydrates decreases postprandial blood glucose by delaying gastric emptying in both healthy adults and adults with type 2 diabetes (Karamanlis et al., 2007; Ma et al., 2009). In adults with prediabetes, consumption of protein prior to carbohydrates decreases postprandial glucose iAUC compared with consuming carbohydrates first for the same meal composition (Shukla et al., 2019). The significantly higher amount of protein consumed with the HP snacks may have delayed gastric emptying and slowed carbohydrate absorption compared with the SP snacks.

Conversely, the peak serum triglycerides concentration was higher with HP snacks than SP snacks, although there were no differences observed between the snack groups for fasting triglycerides or triglycerides iAUC. Alcohol consumption has been previously associated with increased blood triglycerides (Klop et al., 2013). However, as triglycerides only started to peak toward the end of the sample collection, it is likely that only a portion of the overall trajectory was captured.

As alcohol consumption may stimulate appetite and food intake, a focus on the nutritional quality of the foods consumed with alcohol is warranted, particularly in the context of obesogenic environments in populations who regularly consume alcohol. In a qualitative study conducted on alcohol and food, consumers valued pairing foods with wine; however, the pairing of foods with beer was less important (Simone & Steve, 2006). Beer was often consumed with “junk food” and convenience foods.

The development of more palatable, healthier savory snacks continues to grow in response to increasing demand from consumers (Cordeiro et al., 2022). An exploratory study conducted in New Zealand found that nutrition is a factor considered by snack food consumers in addition to the snack's sensory properties when considering snack purchases, with sugar and fat being the two most important nutrients (Forbes et al., 2016). The broader retail availability of palatable snack choices with high nutritional quality provides opportunities for young drinkers to consider swapping these snacks for the usual snacks they consume when drinking alcoholic beverages.

Future research should consider increasing the protein content of the snacks and their acceptability with alcohol consumers and trialing the snacks with different population groups, including clinical populations such as people with prediabetes. The type of alcohol consumed with snack foods should also be varied, as there may be specific associations with the foods that are consumed with beer, wine, and spirits. Although the purpose of this study was to investigate the impact of consuming HP under moderate alcohol drinking conditions, a non‐alcoholic beverage comparator would be useful to determine the impact of alcohol on the intake of HP snacks and SP snacks.

Strengths of this study include that a single‐blind, randomized crossover design was used, with the true aim of the study concealed to minimize altered eating behavior and prevent confounding results. Additionally, the study primarily used retail‐available snack products that may be more generalizable and familiar to participants than formulated snack foods, which can provide pragmatic options for social drinkers. The study was conducted under controlled conditions, including the use of a standardized lunch meal and seating participants away from each other during the testing session. The testing sessions were conducted from the afternoon to the evening to reflect real‐world conditions when alcohol would be more commonly consumed during the day.

However, the study design had limitations that warrant consideration. First, serum cholesterol and serum triglyceride measurements were collected over a short duration of 3 h. This provided an incomplete overview of their trajectories, as was evident for triglycerides. Second, this study did not have a non‐alcoholic beverage condition, and it is unclear the extent that the alcoholic beverage influenced the intake of the snacks. Third, as palatability is an important factor in food intake, the lower palatability ratings of the HP snacks, when compared with the SP snacks, may have influenced snack intake (Brunstrom & Shakeshaft, 2009; de Graaf et al., 2005). Fourth, this study involved cannulation and the measurement of food and alcohol over a few hours. Participants were not familiar with one another but were able to talk to each other across the lab if they wished, and some used handheld devices to pass the time. Participant socialization and screen use may have influenced food intake. Tighter control over these conditions could assist with minimizing the potential influence on food intake, but likewise, leaving subjects to eat alone is also known to impact food intake.

This study suggests that in healthy adults, there is little benefit from simply swapping consumption from SP to HP snacks after a moderate alcohol dose in terms of appetite, food mass, or overall energy intake. However, these findings must be considered in the context of the practical limitations of controlling the conditions in which food intake was measured. The impact of the nudge would be worthy of further investigation, particularly for people with impaired glucose control.

## AUTHOR CONTRIBUTIONS

**Alastair Kwok:** Conceptualization (supporting); data curation (lead); formal analysis (lead); investigation (supporting); methodology (supporting); project administration (lead); writing – original draft (lead); writing – review and editing (equal). **Aimee Dordevic:** Conceptualization (supporting); formal analysis (supporting); investigation (supporting); methodology (supporting); supervision (equal); writing – original draft (supporting); writing – review and editing (equal). **Helen Truby:** Conceptualization (lead); formal analysis (supporting); investigation (lead); methodology (lead); supervision (equal); writing – original draft (supporting); writing – review and editing (equal).

## FUNDING INFORMATION

This work was supported by a Coopers Brewery Foundation community grant. The funders had no influence on the execution of the study, the analysis and interpretation of the data, or the manuscripts and their conclusions. AK is supported by an Australian Government Research Training Program Scholarship.

## CONFLICT OF INTEREST STATEMENT

The authors declare that they do not have any conflicts of interest.

## ETHICS STATEMENT

This study was approved by the Human Research Ethics Committee of Monash University.

## INFORMED CONSENT

Written informed consent was obtained from all study participants.

## Data Availability

The data that support the findings of this study are available from the corresponding author upon reasonable request.
